# Pseudorabies virus in wild swine: current evidence and interpretations from monitoring studies

**DOI:** 10.1186/s12917-026-05661-y

**Published:** 2026-07-02

**Authors:** Thomas Müller, Nicolai Denzin, Conrad M. Freuling

**Affiliations:** 1 Institute for Molecular Virology and Cell Biology, Friedrich-Loeffler-Institut, Südufer 10, Greifswald-Insel Riems, 17493 Germany; 2 Institute for Epidemiology, Friedrich-Loeffler-Institut, Südufer 10, Greifswald-Insel Riems, Germany Germany

**Keywords:** Suid alphaherpesvirus 1, Pseudorabies virus, Aujeszky's disease, Wild swine, Global situation, Monitoring

## Abstract

To synthesize current knowledge on pseudorabies virus (PRV; also known as Aujeszky’s disease virus or *Suid alphaherpesvirus 1*) infections in wild swine, a combined review and meta-analysis was conducted. The study aimed to critically interpret reported PRV seroprevalence data, assess risks for animal health and disease control programs, and inform future monitoring and management strategies. An extensive literature search covering the period from 1970 to 2025 identified 160 serosurveys published between 1979 and 2024, yielding 516 datasets for analysis. Binomial logistic and mixed-effects logistic regression models were used to assess spatio-temporal patterns. The results indicate that PRV is widely established in wild swine populations, particularly in Europe and North America, forming stable endemic cycles largely independent of host density. Persistent spatial heterogeneity and region-specific temporal trends were observed, and once introduced into naïve populations, PRV appears to persist long term, limiting the added value of continued monitoring in long-established endemic areas.

## Introduction

Wild swine (*Sus scrofa*) — members of the Suidae family — are one of the most abundant and also successful invasive species around the world [1]. They have a wide and varied natural distribution range, covering parts of Europe (from Western Europe to Russia), much of Asia, and limited parts of North Africa, although their conspecifics have also been introduced to other continents [2–5]. Their introduced distribution range comprises North America [particularly the southern United States (US) and Hawaii], South America, Australia, New Zealand and some Pacific islands where they occur either as true naïve wild boar, feral populations of domestic origin or as hybrids resulting from interbreeding with domestic pigs [6–8]. The extent of crossbreeding can vary by region, complicating visual classification [9–14]. This often influences whether the animals are classified as introduced wild boar, feral swine, or escaped domestic pigs. While European wild boar have lower genetic diversity compared to their Asian wild boar counterparts [10, 15], about 97% of the invasive feral pigs in the US are estimated to be hybrids [13]. Recent population data from Europe and North America suggest that wild boar and feral pig populations are increasing, expanding way beyond their original ranges and now numbering in the millions [2, 16, 17]. These developments give rise to concerns about increasing negative impacts such as agricultural and biodiversity damage as well as sanitary risks [18–20]. Wild boar and their free-ranging relatives, referred to as wild boars in the context of this study, play a significant role in the potential transmission of infectious diseases to their domesticated form, the domestic pig populations, posing serious risks to animal health and agricultural biosecurity [21]. Despite growing recognition of the importance of wildlife reservoirs in disease epidemiology, often knowledge gaps remain. These include the mechanisms of maintenance of causative agents in wild swine, the impact of host density and landscape features, and the potential for genetic variation or recombination among field strains, cross-species spillover dynamics, and the effectiveness of current monitoring strategies in detecting and predicting outbreaks. As free-ranging animals, wild swine can act as reservoirs and vectors for a range of pathogens, particularly economically important or zoonotic pathogens including African swine fever (ASF) [22, 23], classical swine fever (CSF) [24, 25], Hepatitis E [26, 27], brucellosis [28, 29], tuberculosis [30, 31] and Aujeszky’s disease [32, 33], among others.

Pseudorabies virus (PRV), also known as *Suid alphaherpesvirus 1* (SuHV-1), belongs to the genus Varicellovirus within the subfamily Alphaherpesvirinae of the Herpesviridae family [34] and is a neurotropic DNA-virus responsible for Aujeszky’s disease - a notifiable condition with significant implications for animal health and the swine industry. Although more than 20 countries, primarily in Europe, North America, and Australia have successfully eliminated Aujeszky’s disease from domestic pig populations [35], serological evidence suggests that various PRV variants continue to circulate in free-ranging wild swine populations in North America and Europe. This has raised concerns that wild swine may still serve as a reservoir of infection, potentially threatening disease-free status and complicating ongoing control and eradication efforts in domestic pigs [36]. Since the last global assessment of PRV in wild swine more than 15 years ago, the body of research on the epidemiology, ecology, and transmission dynamics of PRV in wild swine has expanded considerably. Against this background, the present review and meta-analysis aims to synthesize the accumulated and latest knowledge on the occurrence of PRV in wild swine, critically evaluate and interprete reported PRV seroprevalence estimates, assess the potential risks posed to animal health and disease control programs, and, where appropriate, identify key knowledge gaps and research priorities to inform future monitoring, prevention, and management strategies.

## Materials and methods

### Literature search

A systematic literature review was conducted to identify published studies reporting serological surveys of PRV in wild swine. PubMed, Scopus, and bioRxiv were searched for articles published between January 1, 1970, and October 31, 2025, using the following key terms: “pseudorabies” or “Aujeszky’s disease” in combination with “serosurvey” and one or more of the following terms: “wild boar,” “wild swine,” “feral swine,” or “feral hog.” To complement the database searches, additional literature was identified through targeted Google searches conducted in English, Russian, and Spanish to capture reports and publications not indexed in the selected databases. Also, reference lists of retrieved articles were screened iteratively to identify additional relevant studies. This process continued until no further eligible literature was identified. Following full-text review, references were excluded if they were not published in the selected languages or if the target species differed from those defined above.

### Data collection, preparation and processing

Data on the following aspects were collected from the relevant country-specific literature: (i) location of the study area including its size, (ii) period of the seroprevalence study, (iii) sample size, (iv) number of seropositive animals, (v) 95% confidence interval and (vi) serological tests used. Regarding the former, the size of the respective sub-areas, if not already available, was determined for each study based on the area designation from other sources. Individual studies were stratified according to research areas and treated as individual data sets. For studies that only reported percentages of seropositivity, 95% confidence intervals (95% CI) for the serosurvey derived seroprevalence were calculated using the Clopper and Pearson method [37].

### Statistical data analysis

For descriptive purposes, the cumulative PRV seroprevalence on country level was categorized as very low (< 5%), low (5–15%), moderate (15–40%), high (40–70%), and very high (> 70%).

A total of 512 data sets were available for spatio-temporal analysis, for weighted average seroprevalence (total positives divided by total tested) was calculated for descriptive summaries across national studies. Spatial analysis of PRV seroprevalence concentrated on aggregated data from Europe and the US, where the great majority of serological studies were conducted. Pairwise differences in seroprevalence between countries were evaluated using a binomial generalized linear model (GLM) with Tukey-adjusted multiple comparisons. Continent-specific temporal trends were evaluated for Europe, the Americas and Asia using binomial logistic regression models fitted to aggregated study-level data, with sampling year as a continuous predictor and the number of tested animals specified as the binomial denominator. Odds ratios per year and 95% confidence intervals were derived from model coefficients. Model-based predictions were used for visualization, with time scales defined according to the reported study periods in each region. Detailed temporal trends were analyzed for countries and regions with repeated measurements over multiple decades in Europe and the US states using mixed-effects logistic regression models (repeated measures by country) using mid-point of study period as time variable and individual study records (not aggregated) as study units. After exclusion of records lacking temporal information or sample size, 489 and 400 datasets were included in continental and detailed temporal trend analyses, respectively.

The correlation between wild boar density and cumulative PRV seroprevalence was assessed for Europe as an illustrative example. To this end, population gridded data were derived from the ENETWILD wild boar density model developed for the European Food Safety Authority (EFSA, DOI: https://doi.org/10.5281/zenodo.10784987). Only seroprevalence studies since 2010, representing 120 data sets, were considered in order to reasonably reflect the most recent estimates of wild boar density. Centroids for serological sampling locations were derived from Datawrapper and buffered by 50 km. The average wild boar density was calculated and assigned to the respective zones. GIS analyses were performed in QGIS-Version 3.22.0-Białowieża (https://qgis.org/). To contextualize sampling effort across wildlife serosurveys, sampling density (samples·km⁻²) was calculated as sample size divided by study area and summarized by study scale (< 1,000 km²; 1,000–10,000 km²; 10,000–100,000 km²; >100,000 km²) using medians and IQRs. For this purpose, information on the size of the study area was taken from the data in the published study and, where this was not available, derived from the name of the study area or the broken-down regional districts or municipalities from public sources.

### Data processing and availability

All simulations, distribution fitting and regression analyses were conducted using the open-source software R/Python (pandas). For spatio-temporal analysis, DeepSeek (OpenAI) was used for minor code refinement. All analyses, statistical procedures, and interpretations were conducted and independently verified by the authors. Graphical visualizations were realized using standard plotting libraries including Datawrapper (www.datawrapper.com/) or GraphPad Prism version 8.0.0 for Windows (GraphPad Software, San Diego, California USA, www.graphpad.com). All datasets and metadata used in the analysis are publicly available on the Zenodo repository at https://doi.org/10.5281/zenodo.19108588

## Results and discussion

### Occurrence of PRV infections in wild swine

Monitoring wild swine populations is an essential component of disease prevention and containment strategies within the swine industry [21]. In contrast to other viral diseases, PRV infections in wild swine are often asymptomatic or result in only very mild clinical signs, which facilitates undetected virus persistence and transmission and renders clinical detection complicated [32, 38]. On the other hand, herpesvirus infections are typically defined by their ability to establish lifelong latency, where the virus remains dormant in the host after the initial infection, while the immune system continuously suppresses its reactivation [39, 40]. This pathogenic hallmark makes the detection of virus specific antibodies a very good proxy for the occurrence and spread of PRV infections in populations of domestic pigs as well as of wild swine [41].

**Fig. 1:**
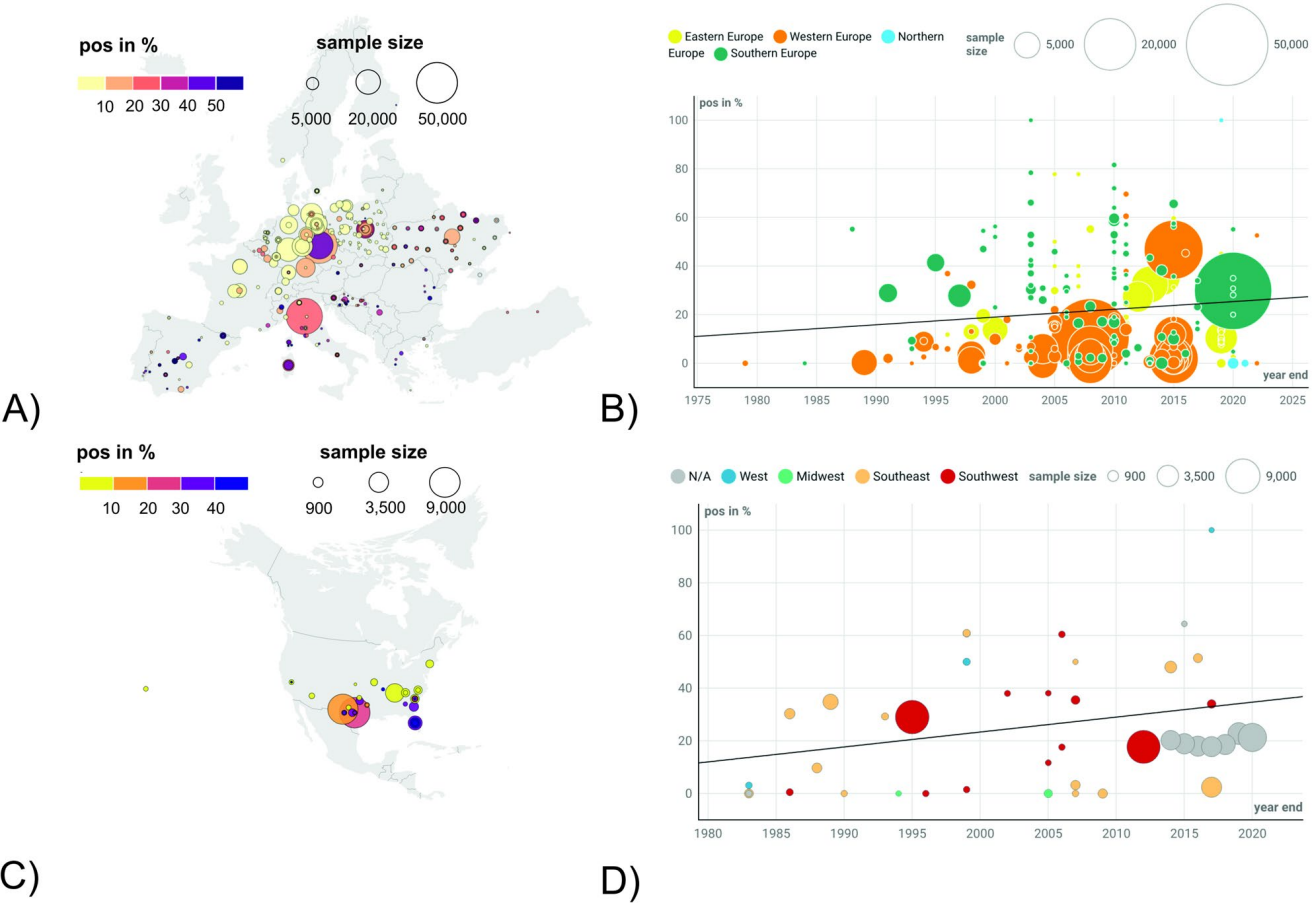
Visualization of PRV serosurvey locations and seroprevalences in wild swine in Europe and North America. Panels **A** and **C** show the study locations, while panels **B** and **D** display the corresponding region-specific time periods of PRV serosurveys conducted in wild boar and feral swine populations between 1979 and 2024, based on the data presented in Table 1. Circle size reflects the sample size used to estimate seroprevalence, and the colour scale indicates the respective seroprevalence ranges. Graphs were created using Datawrapper (www.datawrapper.com) and combined in CorelDRAW 2017 (Corel Corporation)

Serological studies on PRV in wild swine are mostly conducted in response to the prevailing disease status in the domestic pig population, with their scope often shaped by public concern and available funding sources [42]. Previous reviews of wild swine-mediated PRV reported on serosurveys in 19 countries (e.g. in Europe and the US) covering the period between 1979 and 2014 [32, 42, 43]. In the meantime, additional studies have been published in international literature or are now available in digital form from other sources. As a result, between 1979 and 2024, a total of 161 scientific studies and reports on PRV seroprevalence in wild swine were published, covering 34 countries across four continents, with the great majority originating from Europe and the US (Table 1; Fig. 1A, C).

**Table 1:** Global overview of country-level PRV serosurveys (1979–2024) with study details

country			wild boar		time period		aggregated				
	No. of studies/ reports	study area in km²	free- range	farmed	from	to	sample size	sero- prevalence in %	sero- prevalence level	new data this review	reference
Argentina	1	2,500	x		2009	2009	75	41.3	high		[44]
Austria	3	19,186	x	x	2010	2011	631	43.3	high	x	[45–47]
Belgium	2	25,669	x		2004	2024	3,200	18.1	moderate	x	[48, 49]
Brazil	4	799,229	x	x	1998	2018	748	42.9	high		[50–53]
Colombia	1	38	x		2009	2009	15	0	very low	x	[54]
Croatia	4	56,594	x		1999	2020	1,720	57.1	high	x	[55–58]
Czech Republic	1	78,870	x		2004	2005	338	29.9	moderate		[59]
Finland	1	15,000	x		2016	2019	235	0.4	very low	x	[60]
Former Yugoslavia	1	255,804	x		1988	1988	96	55.2	high		[61]
France	8	551,695	x	x	1979	2022	20,808	6.1	low	x	[62–69]
Germany	12	357,592	x	x	1985	2015	210,342	10.9	moderate	x	[70–81]
Greece	2	21,396	x	x	2006	2013	524	34	moderate	x	[82, 83]
Iran	1	?	x		?	?	28	42.8	high		[84]
Italy	17	169,719	x	x	1983	2020	62,335	28.3	moderate	x	[85–100]
Japan	4	278,827	x		2003	2021	1,838	3.1	very low	x	[101–104]
Lithuania	2	65,300	x		2001	2004	59	3.4	very low	x	[105, 106]
Mexico	1	1,125	x	x	2016	2016	42	0	very low	x	[107]
Netherlands	4	1,363	x		1994	2001	1,728	0.2	very low		[108–112]
New Zealand	1	268,021	x		1993	1995	455	0	very low	x	[113]
Norway	1	10,500	x		2021	2021	294	0	very low	x	[114]
Poland	5	322,575	x		1994	2024	60,728	17.4	moderate	x	[115–119]
Portugal	2	5,405	x		2010	2015	221	29.4	moderate	x	[120, 121]
Romania	2	13,936	x		2008	2015	541	51.8	high	x	[122, 123]
Russia	2	1,094,533	x		2002	2007	122	36.1	moderate		[124–126]
Serbia	2	88,499	x		2013	2015	833	39.1	moderate	x	[127–129]
Slovakia	4	49,035	x		2014	2019	348	36.7	moderate	x	[130, 131]
Slovenia	4	20,273	x		2003	2011	2,908	22.5	moderate	x	[132–135]
South Korea	5	10,0210	x		2010	2023	2,764	4.8	very low	x	[136–140]
Spain	12	361,187	x	x	1999	2015	7,375	37.3	moderate	x	[33, 121, 141–150]
Sweden	7	13,968	x		2006	2023	2,069	0	very low	x	[151–157]
Switzerland	4	41,285	x		2001	2013	4,145	2.4	very low		[158, 159]
Tunisia	1	11,807	x		1993	1995	71	63.4	high		[160]
Turkey	1	16,154	x		2012	2012	93	23.7	moderate	x	[161]
Ukraine	5	603,628	x		2001	2020	21,172	20.4	moderate	x	[125, 162–165]
United States	34	2,520,890	x	x	1979	2019	53,738	24	moderate	x	[20, 166–196]
total	15 4	8,241,813			1979	202 4	462,836				

These studies include data on free-ranging and farmed wild swine. As part of these studies, serum samples from approximately 462,800 animals were collected across various regions, spanning a total area of over 8.3 million km² (Table 1). These global studies demonstrated regional variation in the seroprevalence of PRV-specific antibodies (Fig. 1), documenting evidence for the endemic circulation of PRV variants in wild swine populations in Europe, parts of North and South America, parts of Asia, and even Northern Africa (Table 1). For countries bordering regions where PRV is endemic in wild swine populations, the absence of publicly available data does not necessarily indicate that they are unaffected. On the other hand, PRV endemicity in wild swine may also be inferred from molecular detection or virus isolation, which provide direct evidence of active or recent infection, in contrast to serological findings that primarily indicate virus exposure; examples are Hungary and the Slovak Republic [197]. There is evidence that SuHV-1 has been investigated serologically in Hungarian wild boar populations, unfortunately however detailed results and bibliographic metadata are not publicly accessible.

No peer-reviewed publications or traceable reports, however, are available that report PRV seroprevalence or infection in wild swine from Asian countries other than South Korea and Japan. There is one notable survey in Tibetan pigs which are primarily found in the Qinghai-Tibet Plateau region of China [198, 199], that found a 15.8% PRV seroprevalence via ELISA, but this refers to domestic plateau pigs, not truly feral animals [200]. Although African suids such as the Warthog (*Phacochoerus spp.)*, Bushpig (*Potamochoerus larvatus*), Red River Hog (*Potamochoerus porcus*) and the Giant Forest Hog (*Hylochoerus meinertzhageni*) [201] can become infected with PRV, there are no published experimental or field studies confirming real-world infections in these species. Otherwise serosurveys in African wild pig populations have never been systematically envisaged. Similarly, serological surveys of peccaries (New World pigs) in North and South America [201] have not yet revealed any significant seroprevalence that would indicate persistent PRV infections in these species [54, 202–204]. Thus, there is reason to belief that, unlike wild swine, peccaries do not appear to be a reservoir for SuHV-1.

### Spatial heterogeneity

As per design, the great majority of published PRV serosurveys in wild swine populations are cross-sectional studies or serial cross-sectional studies, in which PRV seroprevalence data on a given population are collected either at a single point in time or over a very short, defined period or data collection spans several years. As a result, the proportion of PRV-positive animals reported are aggregated seroprevalences. This measure reflects the percentage of animals which possess PRV-specific antibodies at any point during a defined timeframe or up to a certain time (from baseline to current time), thereby providing evidence of exposure of a portion of the wild swine population to the pathogen, i.e. PRV, or even long-term circulation [205, 206]. Hence, these studies provide snapshots on current or past PRV infection prevalence rather than incidence [207]. This allows comparisons between regions or populations to identify hotspots or areas of higher exposure risk, although incidence rates would be far more appropriate but are difficult to determine.

Based on available data sets (*n* = 516), the overall aggregated PRV seroprevalence in wild swine populations shows substantial variation, with mean values ranging from 0% to 63% across global studies (Table 1). Unfortunately, there are no universal epidemiological thresholds to define different categories of seroprevalence in (wild) animal populations. Often explicit, study-specific descriptive categories for interpretation are used that are not meant as biological rules, but rather as communication tools. When such defined descriptive ranges are applied to classify country-specific average PRV seroprevalence levels in wild swine populations across all national studies over the entire study period (1979–2024), 32.3% of countries fall within the very low (< 5%), 5.9% within the low (5–15%) and 41.2% within the moderate category (15–40%). In contrast, 20.6% of countries exhibit high seroprevalence levels (40–70%), while no country reports a very high PRV seroprevalence (> 70%) (Table 1).

If data sets from Europe and the US are aggregated (*n* = 421) for each country/state per decade and weighted seroprevalence for each period is calculated, the spatial analysis reveals greater spatial heterogeneity (Table 2). Based on comparable sample sizes and study designs, PRV seroprevalences in both European and US regions show marked spatiotemporal variation. In total, 74.3% and 75.6% of regional pairs in Europe and the US, respectively, showed significant differences (*p* < 0.05) between regions. In both geographical areas, several countries or regions exhibited consistently moderate to high PRV seroprevalence across multiple decades, consistent with sustained virus circulation and established endemicity. In contrast, for other areas consistently low seroprevalence levels were found, which may indicate limited circulation or a more recent introduction of PRV into the wild swine population (Table 2). Interestingly, in both Europe and the US, a north–south gradient in PRV seroprevalence in wild boar populations is observed. In general, aggregating datasets smooths some variability and highlights overall trends but in turn still may mask important sub-national, sub-regional, or year-to-year patterns.

**Table 2:** Weighted average PRV seroprevalence (%) in wild swine by country/region and decade for Europe and the US (only countries with a cumulative sample size > 100)

Europe						United States				
Country	1980s	1990s	2000s	2010s	2020s	Region	1980s	1990s	2000s	2010s
Austria	-	-	-	43.3	-	Arizona	0.5	-	-	-
Belgium	-	-	17.2	27	-	California	28.2	-	3.1	-
Croatia	-	27.4	33.6	27.3	-	Florida	34.8	51.4	48	51.4
Czech Republic	-	-	29.9	-	-	Georgia	30.3	-	-	-
Finland	-	-	-	0.4	-	Hawaii	0	-	-	-
France	0	2.8	8.3	25.2	23.4	Kansas	-	0	-	-
Germany	0.4	4.3	9.1	13.4	-	Missouri	-	-	0	-
Greece	-	-	40.8	29.7	-	Multistate	-	-	17.7	21.8
Italy	28.9	23	12.6	30.5	-	North Carolina	-	-	0	-
Netherlands	-	0.2	0	-	-	Oklahoma	30.3	-	-	34
Poland	-	7.1	1.3	31.4	32.2	South Carolina	29.3	60.9	50	9.7
Portugal	-	-	-	34.6	-	Tennessee	-	0	3.2	2.4
Romania	-	-	55.2	46.3	-	Texas	33.2	29	32.4	31.4
Russia	-	-	36.4	-	-					
Serbia	-	-	-	39.3	-					
Slovakia	-	-	-	36.7	-					
Slovenia	-	-	21.5	45.1	-					
Spain	-	37.5	38.3	2.8	-					
Sweden	-	-	0.1	0	0					
Switzerland	-	-	3.7	0.6	-					
Ukraine	-	-	20.5	19.2	-					

### Temporal trends in PRV seroprevalence in wild swine

When broken down geographically, temporal trends in PRV seroprevalence differed markedly between continents, underscoring the importance of region-specific epidemiological contexts when interpreting long-term monitoring data from wild swine populations. For example, the overall PRV seroprevalence in European wild boar populations showed a statistically significant increase of 2.2% per year (*p* < 0.001) over the past five decades. The fitted model suggests a progressive rise in the odds of seropositivity by 2.2% annually continent-wide (Fig. 1B), which may reflect sustained long-term endemic persistence and cumulative exposure of PRV within European wild boar populations. Mixed-effects model projections suggest that European PRV seroprevalence may reach ~ 20% by 2030 if current trends persist. However, substantial between-country variability was observed, with highly significant differences in seroprevalence rates among country–decade combinations (χ² ≈ 58,742, df = 23, *p* < 0.000001). While some European countries with repeated measurements over multiple decades exhibited relatively stable trends or maintained near-zero seroprevalence (Table 2), other countries including France, Poland, Germany, Belgium and Slovenia showed historically low seroprevalence levels followed by more recent increases. These increases were statistically significant (*p* < 0.05) only for the first three countries and corresponded to annual percentage changes ranging from approximately 1.5% to 4.2%, indicative of slow to moderate growth and suggesting a genuine expansion of PRV in these regions. Spain displayed a more volatile pattern, with an apparent statistically significant (*p* < 0.05) decline from approximately 38% in the 2000s to around 3% in the 2010s. However, this sharp decrease is likely an artefact, as the data for the 2010s are dominated by a single large study (*n* = 971) conducted in two locations (Ciudad Real and Toledo) that reported zero prevalence [150]. Excluding this study results in a largely stable trend over time, underscoring the risk of drawing conclusions from single, potentially unrepresentative large datasets (Table 2).

In contrast in the Americas, PRV seroprevalence in feral swine populations appeared to exhibite a relatively stable to slightly decreasing temporal trend, with odds of seropositivity remaining broadly constant over time. However, the data from the Americas were too heterogeneous, as they included data from Mexico and several South American countries in addition to the US (Table 1), and comparatively sparse, resulting in more limited temporal coverage than in Europe. Looking at the US alone, a similar increase in PRV seroprevalence can be observed there as in Europe (Fig. 1D), with states such as Tennessee, Oklahoma, and Florida showing the largest increases in seroprevalence, ranging from 0.9 to 4.6% points per decade, while the temporal analysis for Texas, California and South Carolina indicated a minimal decline in PRV seroprevalence; however, none of these trends regarding the latter states were statistically significant (Table 2).

Similar to the situation in America, the uneven availability of data across time and geographical area (mainly South Korea and Japan, Table 1) made it impossible to derive general trends in PRV seroprevalence for wild boar populations in Asia. Although early studies in Asia reported a relatively moderate to high PRV seroprevalence for some countries such as Iran and Turkiye, later studies indicate significantly lower overall levels of exposure, with some variability at the regional level (Japan, South Korea).

### Wild swine population density and seroprevalence

**Fig. 2:**
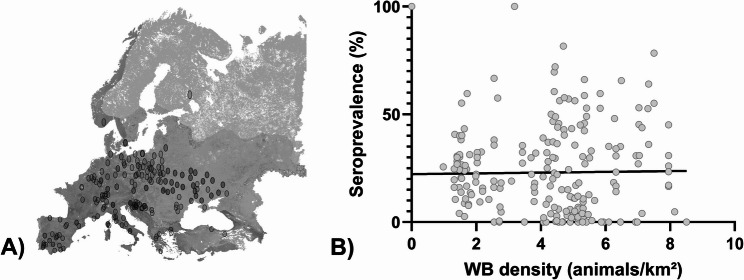
**A** Map of Europe showing wild boar density derived from the ENETWILD wild boar density model developed for the European Food Safety Authority (EFSA, DOI: https://doi.org/10.5281/zenodo.10784987), with points indicating serological sampling locations. Only seroprevalence studies since 2010 were considered in order to reasonably reflect the most recent estimates of wild boar density. **B** Scatterplot illustrating the association between wild boar (WB) density (animals/km²) and PRV seroprevalence (%). Each point represents a single study site; the solid line denotes a simple linear regression

Populations of wild swine in Europe and the US have been steadily increasing in recent decades [18, 208, 209]. Studies estimating the population size of invasive wild swine in the US states have shown that feral pigs now occur in at least 35 US states, with major concentrations in Texas, Oklahoma, Louisiana, Georgia, Florida, and New Mexico. Nearly all states where wild pigs are present have experienced population increases, resulting in an estimated nationwide population of approximately 6.9 million animals [17]. Likewise, for Europe the most recent high-resolution mapping and density models that combine hunting statistics, occurrence data, and environmental predictors from across the continent place the wild boar population at between 13.5 and 19.6 million individuals in the core wild boar range before the annual hunting season [210]. As these are just robust estimates, the real population size of wild boar in Europe might even be higher. Although estimates may vary substantially for some European countries and may require adjustment where local factors strongly influence wild boar populations, the resulting outputs can nevertheless be used to assess the risk of disease spread and the potential effects of management measures [210]. Because European serosurveys cover a 45-year time span, whereas population data are only available for 2025, this poses methodological challenges. However, when recent (2025) gridded wild boar population data for Europe [210] are exemplarily applied to PRV seroprevalence data since 2010, no correlation is observed between regional cumulative PRV seroprevalence and wild boar density (Fig. 2). While this approach is not optimal due to temporal mismatches in the datasets, it nevertheless provides a rough approximation and suggests that the relationship between PRV seroprevalence and host density is likely complex and not adequately captured by density estimates alone. Presumably factors other than host density may play a more important role in shaping PRV seroprevalence patterns.

### Factors Influencing PRV seroprevalence estimates in wild swine

**Fig. 3:**
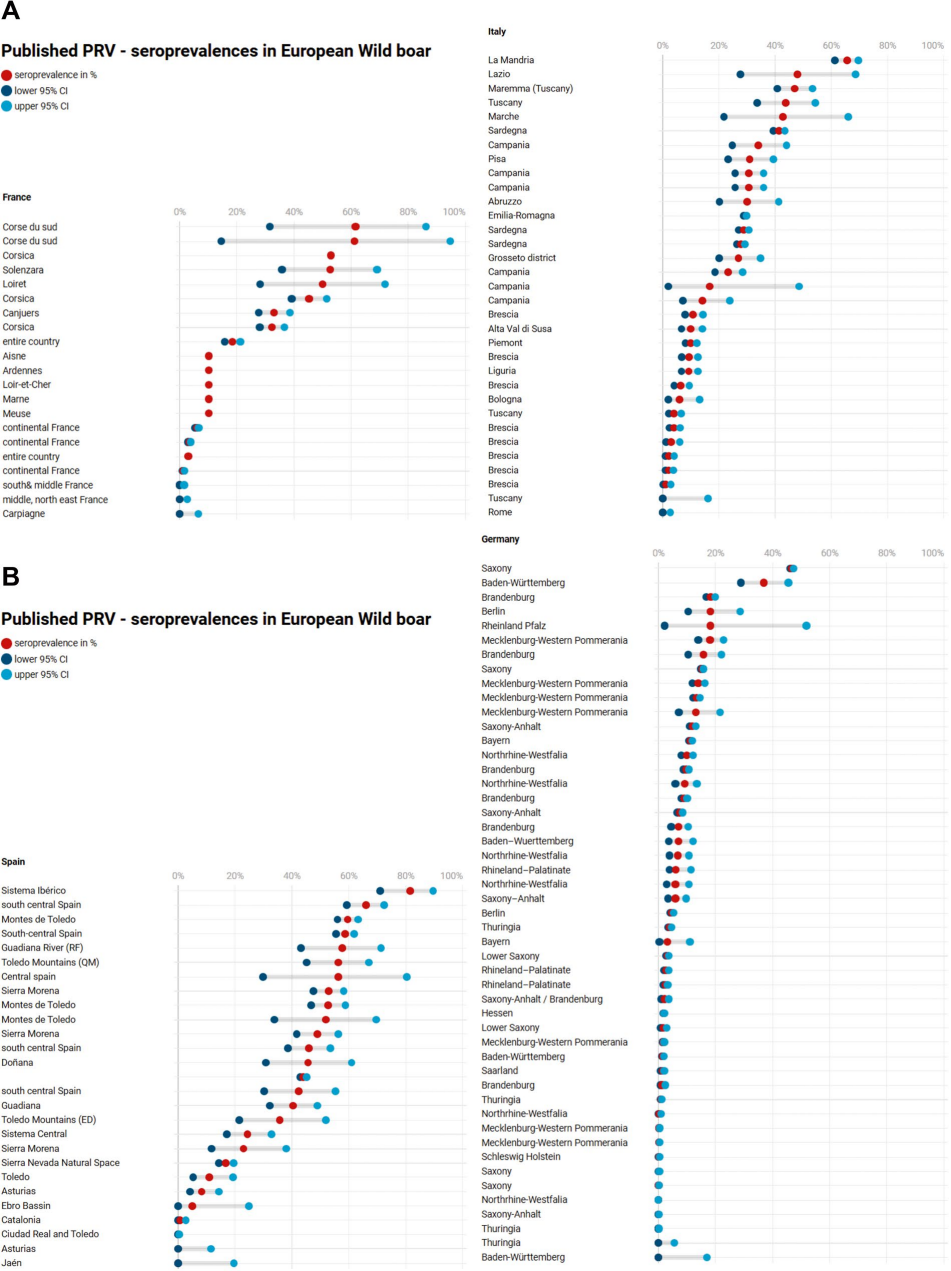
PRV point seroprevalence (red) with 95% confidence intervals (blue) in wild swine populations across region-specific time periods, exemplified for France and Italy **A** and Germany and Spain (**B**). Regions are ordered from highest to lowest seroprevalence. Repeated occurrences of country-specific regions indicate different studies conducted at distinct time periods. Graphs were created using Datawrapper (2025) https://www.datawrapper.de

As with other wildlife diseases interpreting this data is challenging as it is limited by various factors that may potentially distort PRV seroprevalence estimates [205, 211]. For example, if (cumulative) seroprevalence is reported solely as the percentage of positive samples (point estimate), it may give a misleading impression of the actual seroprevalence. When the 95% confidence intervals (95% CIs) are calculated for all reported studies, they offer a more reliable estimate by presenting a range that likely contains the true PRV seroprevalence in the wild swine population. These intervals account for sample size and variability, with wider CIs indicating smaller sample sizes and greater uncertainty, and narrower CIs reflecting larger sample sizes and increased confidence in the estimate. In any case, applying CIs helps avoid overinterpreting seroprevalence in studies with small or uneven sample sizes and allows comparison of PRV seroprevalence across regions during a defined period (Fig. 3A,B).

Differences in assay performance (e.g., sensitivity, specificity, and time to antibody detection) between virus neutralisation tests, glycoprotein B (gB) and E (gE) ELISAs, and latex agglutination tests (LAT) have been demonstrated, which can lead to variation in estimated seroprevalence across studies [212]. ELISAs are more sensitive than traditional serum neutralisation test (SNT), while LATs have shown variable performance compared with ELISAs and SNT, with differences in sensitivity and timing of antibody detection depending on study design and infection stage [213]. Hence, across studies, differences of ± 5–15% (or more) can arise purely from test choice. Therefore, as a general principle, when sensitivity (Se) and specificity (Sp) estimates are derived from validation studies as is typically the case for serological assays, the “true” 95% confidence interval should incorporate the additional sampling variability associated with Se and Sp [214]. Information on diagnostic assays was available for 152 studies: the SNT, LAT, and ELISA were used exclusively in 16, 7, and 126 studies, respectively. Ten studies applied a combination of different assays. With regard to ELISAs specifically, at least 11 different commercial PRV gB/gE kits and three unspecified ELISA tests were employed. However, because information on Sn and Sp of most serological assays was difficult to obtain, adjusting the 95% CIs for all studies was not possible.

Although ELISAs are generally less susceptible to sample degradation than serum neutralization tests, severe hemolysis and post-mortem changes may affect test performance. However, given that most studies included in this review (152/160) relied on samples from hunter-harvested wild swine and predominantly used ELISA-based assays (*n* = 126), sample quality is unlikely to represent a major source of bias or explain regional differences in seroprevalence estimates. In addition, mutations within the gE protein could theoretically affect the sensitivity of gE-based serological assays [215]. However, such effects have not been demonstrated for the characteristic gE insertions reported in currently circulating PRV variants [216]. Fortunately, the diagnostic tests are highly PRV specific, as there is no cross-reactivity between PRV and other suid herpesviruses. The latter viruses like suid herpes virus 2 and 3 are betaherpesviruses [35], which reduces the likelihood of significant cross-reactivitiy due to genetic divergence, hence PRV diagnostic tests are not affected by these [41]. Yet undiscovered or uncharacterized herpesviruses in feral/wild boar populations could pose a risk for serological cross-reactivity, however this remains speculative.

Sample size for example is a critical factor, too, that varies widely between studies, regions, and time periods (Fig. 1). The cumulative sample sizes reported in national serosurveys differ greatly (Table 1). Among the 34 countries where PRV serosurveys were conducted, seven (20.5%) had cumulative sample sizes below 100; seven (20.5%) had between 101 and 500; five (14.7%) between 501 and 1,000; eight (23.5%) between 1,001 and 5,000; one (2.9%) between 5,001 and 10,000; and five (14.7%) between 20,001 and 40,000. Germany reported the highest cumulative sample size, with over 210,000 animals tested between 1985 and 2015 (Table 1). One problem associated with high sample size is that they are more likely to produce statistically significant differences, even when the true difference is small. With sample sizes in the thousands across multiple decades, which apply to countries including Germany, Italy, Poland, Ukraine and France even small absolute differences in seroprevalence (2–5% points) can reach statistical significance; therefore, effect sizes and confidence intervals have to be emphasized over p-values.

Apart from sample size and Sn and Sp of diagnostic tests, other factors may also have an impact on the results obtained. For example, temporal and geographic variations in sample collection can affect comparability. Wild boar serosurveys frequently rely on samples obtained from hunter-harvested individuals. This introduces opportunistic or convenience sampling, which may not accurately represent the broader population due to biases in how and which animals are sampled [211], hence sampling bias toward certain age classes, sex or regions can distort prevalence estimates.

**Fig. 4:**
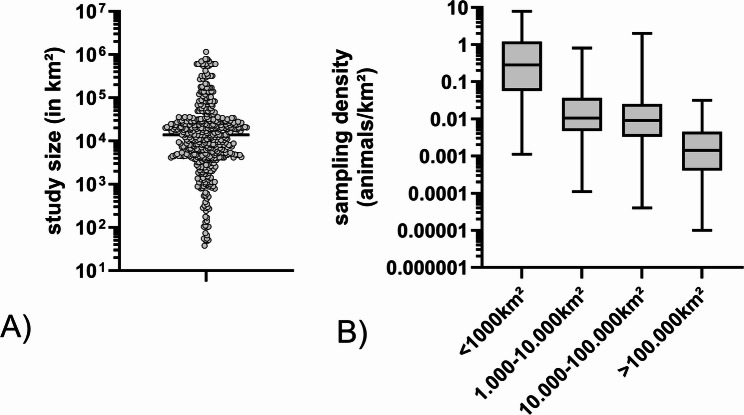
Study size and sampling density (GraphPad Prism version 8.0.0). **A** Size of study areas from all data sets analysed (n 474). **B** Box plot showing sampling density (samples per km², log scale) used in PRV wild swine serosurveys (Table 1) by study scale. Boxes represent the interquartile range (IQR) with median indicated; whiskers extend to 1.5× IQR. Sampling density decreases systematically from study areas < 1,000 km² to > 100,000 km²

Another issue associated with convenience sampling is spatial (cluster) sampling bias [217]. Across the published wildlife serosurveys, sampling density varied by study scale. In the compiled dataset (*n* = 474), mean sampling densities were 1.13, 0.04, 0.06, and 0.004 samples/km² for study areas < 1,000 km² (*n* = 36), 1,000–10,000 km² (*n* = 159), 10,000–100,000 km² (*n* = 225), and > 100,000 km² (*n* = 54), respectively, corresponding to approximately one sample per 0.9, 25, 17, and 250 km² (Fig. 4). Since precise information on the size of the study areas was rarely available in published studies, the estimates derived from these data should be considered approximate. Moreover, for most studies no precise geocoordinates of sampled wild boar were reported, making it unclear whether sampling covered the entire study area or only parts of it. Consequently, some degree of bias related to sampling intensity must be assumed, as the defined study areas may exceed the areas actually sampled. Hence, this analysis does not assume homogeneous animal density or uniform sampling probability within study areas and does not evaluate sampling adequacy for prevalence estimation; rather, it provides a descriptive overview of sampling intensity as reported in the literature. So the variability reflects differences in study objectives and spatial scale rather than a single optimal sampling density.

In wildlife disease serosurveys, sample size is typically determined by monitoring objectives (e.g., detection or prevalence estimation) and target confidence/precision under an assumed sampling design, rather than by geographic area alone; consequently, studies using similar design targets often converge on similar sample sizes, which implies a relatively narrow range of sampling densities at national scale [218]. Because the overall population is typically too large or widespread for simple random sampling, often samples from only a subset of geographically accessible areas, such as specific hunting districts or forest regions are collected and then extrapolated to a greater area. This applies especially to studies with a relatively low sample size compared to the size of the study area. As a result, this cluster sampling may not represent the entire population and may require statistical adjustments to account for the resulting design effect to avoid under- or overestimating variance [217]. Also, for period prevalence, long time frames may mask fluctuations in disease prevalence, underestimating or overestimating peaks of infection [205]. Additionally, wild swine have the highest turnover rates in proportion to their body size compared to other ungulates [2, 219]. Cumulative PRV seroprevalences may thus overrepresent historical exposure relative to the current situation.

### Risk of PRV spillover into domestic pigs and animals

The wide-ranging movements and interactions of wild swine with both natural environments and areas near farms increase the likelihood of direct or indirect contact with domestic pigs. This interface creates opportunities for disease spillover that can have severe consequences for the swine industry [21]. As expected, given the very close genetic relationship between wild swine and their domesticated conspecifics, experimental studies have demonstrated that PRV can, in principle, readily cross from wild swine to domestic swine and vice versa [38, 220]. In fact, about 6 to 18.7% of wild swine in Europe and the US were found to shed PRV in nasal, oral, and genital secretions at a given time point suggesting that transmission via direct (oronasal or venereal) routes of transmission among infected wild swine and to other susceptible animals is possible under field conditions [189, 190]. However, the epidemiological reality appears to be different. Despite the widespread distribution of PRV infections in wild boar populations, only sporadic spillover infections have been documented in confined domestic pigs. This apparent dichotomy, i.e. high PRV prevalence in wild swine alongside successful disease control or freedom from disease in domestic pigs, is consistent with observations from many European countries, where wild boar continue to act as a wildlife reservoir despite effective control measures in domestic pig populations. In Europe, for example, France has reported occasional spillover outbreaks of Aujeszky’s disease each year in free-range pig farms or farmed wild boar in the south or middle of the country since 2019; similarly, Hungary reported outbreaks in 2022. The probable origin was reported to be contact with wild boar. Such incidents are thought to be associated with traditional pig husbandry practices or insufficient biosecurity [221–226], with transmissions mainly occuring via direct contact to wild boar or through venereal infections in the case of free-range pigs [220]. Indirect evidence has been reported from Belgium and Croatia, where genetic characterization of PRV revealed that that some isolates with identical gC sequences occurred in both populations, suggesting epidemiological links and highlighting the risk of potential spillover into domestic pigs [227, 228]. In the US, commercial pig herds have been free since 2004, however, public documents commonly acknowledge sporadic domestic incidents and explicitly link risk to possible feral swine contact [185], while detailed, confirmed “feral → domestic” source attribution (with sequencing/traceback) are scarce. While such outbreaks previously resulted in the suspension of Aujeszky’s disease–free status, this is no longer the case [225, 226]. This contrasts with African swine fever (ASF) and classical swine fever (CSF), which are known to exert substantial infection pressure on nearby pig farms [229]. Interestingly, no cases of transmission from wild boar to other farm animals associated with grazing have been reported. In contrast, there are frequent reports of occasional spillover infections in hunting dogs and other wild carnivores, for which PRV is highly neurotropic, resulting from direct contact due to traditional hunting practices and potentially indirect (through carcass consumption) routes of infection, respectively (for review see [230]). These observations suggest that, although interfaces between wild swine, farm animals, companion animals, and hunting dogs do exist, the prvailing PrV variants in wild swine populations may be less contagious under natural conditions than previously assumed.

## Conclusions

Despite the predominance of data from Europe and the US States, the available evidence indicates that, aside from a few exceptions, PRV has become firmly established in wild swine populations wherever these hosts occur, forming stable cycles of endemicity at a supra-regional scale that appear largely independent of local wild swine density. The observed seroprevalence patterns are therefore not random but reflect pronounced and persistent spatial heterogeneity, with certain countries and regions consistently exhibiting higher odds of seropositivity over multiple decades. At the same time, marked differences in temporal trends between continents point to the influence of biological and region-specific ecological, management, and epidemiological factors shaping PRV dynamics. The data further suggest that PRV is likely to continue expanding geographically; once introduced into previously naïve wild swine populations, affected areas tend to remain persistently infected, largely irrespective of subsequent local fluctuations in seroprevalence. Consequently, continued monitoring in long-established endemic regions is unlikely to yield substantial new epidemiological information or insights. Monitoring efforts should therefore prioritise currently seronegative regions or areas that have not yet been studied by using serological assays with a high diagnostic Sn and Sp.

Importantly, in contrast to several other swine diseases, there is strong evidence that PRV circulation in wild boar does not pose a substantial risk to domestic pig populations provided that strict biosecurity measures are maintained and traditional free-range rearing of domestic pigs in certain regions is strictly monitored. Taken together, these findings emphasise the need for geographically tailored monitoring, risk assessment, and management strategies to adequately interpret and address long-term PRV dynamics in wild swine populations.

## Data Availability

The original data supporting the findings of this study are available on the Zenodo repository at 10.5281/zenodo.19108588 .

## Abbreviations

ASF: African swine fever
CSF: classical swine fever
EFSA: European Food Safety Agency
GLM: generalized linear model
PRV: pseudorabies virus
SuHV: Suid alphaherpesvirus 1
US: United States
